# A simple hepatic cyst with elevated serum and cyst fluid CA19-9 levels: a case report

**DOI:** 10.1186/1752-1947-2-329

**Published:** 2008-10-14

**Authors:** Hidekatsu Yanai, Norio Tada

**Affiliations:** 1 Department of Internal Medicine, Division of General Medicine, Kashiwa Hospital, The Jikei University School of Medicine, 163-1, Kashiwashita, Kashiwa, Chiba 277-8567, Japan

## Abstract

**Introduction:**

Simple hepatic cysts rarely cause symptoms, however, occasionally they become symptomatic due to mass effect, rupture, hemorrhage, and infection. We report a patient with a large hepatic cyst with elevated serum and cyst fluid CA19-9 levels. We studied serum and cyst fluid CA19-9 levels in this patient, before and after the intracystic instillation of minocycline hydrochloride.

**Case presentation:**

A 76-year-old Japanese woman was diagnosed as having an infected hepatic cyst, by physical examination and enhanced abdominal computed tomography. Serum (170 U/ml; reference: < 37 U/ml) and hepatic cyst fluid (371 U/ml) CA19-9 levels were elevated. After the intracystic instillation of minocycline hydrochloride, necrotic cells in the cyst were drained, and it totally collapsed after 1 week. Cyst fluid CA19-9 levels increased remarkably after the intracystic instillation of minocycline hydrochloride, while serum CA19-9 levels decreased significantly.

**Conclusion:**

Our study is the first report to reveal the influence of intracystic instillation of minocycline hydrochloride on serum and cyst fluid CA19-9 levels in a patient with a simple hepatic cyst.

## Introduction

Benign hepatic cysts are commonly observed in the general population, however, they rarely cause symptoms. Simple hepatic cysts are generally stable in size over time, but may grow slowly and occasionally become symptomatic due to mass effect, rupture, hemorrhage, and infection [1]. We report a patient with a large hepatic cyst with elevated serum and cyst fluid CA19-9 levels. Further, we studied serum and cyst fluid CA19-9 levels in this patient, before and after intracystic instillation of minocycline hydrochloride.

## Case presentation

**Figure 1:**
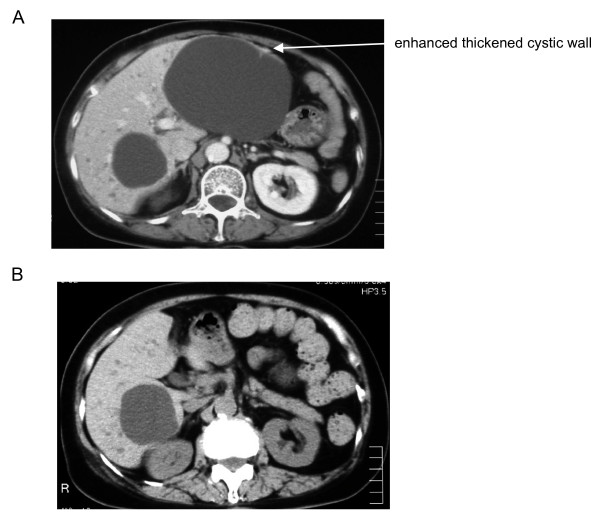
**A) Enhanced abdominal computed tomography before a percutaneous transhepatic drainage of the hepatic cyst.** B) Abdominal computed tomography at 1 week after the intracystic instillation of minocycline hydrochloride.

A 76-year-old Japanese woman was admitted because of fever and chill. Physical examination revealed percussion tenderness in the right upper quadrant. She had previously been diagnosed as having a large simple hepatic cyst and elevated serum CA19-9 levels. Laboratory examination showed increased serum levels of C-reactive protein (CRP) (14.5 mg/dl; reference: < 0.3 mg/dl) and CA19-9 (170 U/ml; reference: < 37 U/ml), and *Escherichia coli *were cultured from blood. Enhanced abdominal computed tomography (CT) showed a large hepatic cyst with partially enhanced thickened cystic wall (Figure 1A). From this and the tenderness to touch in her right upper quadrant, we diagnosed an infected hepatic cyst [2], and started antibiotic therapy using β-lactamase inhibitors.

**Figure 2:**
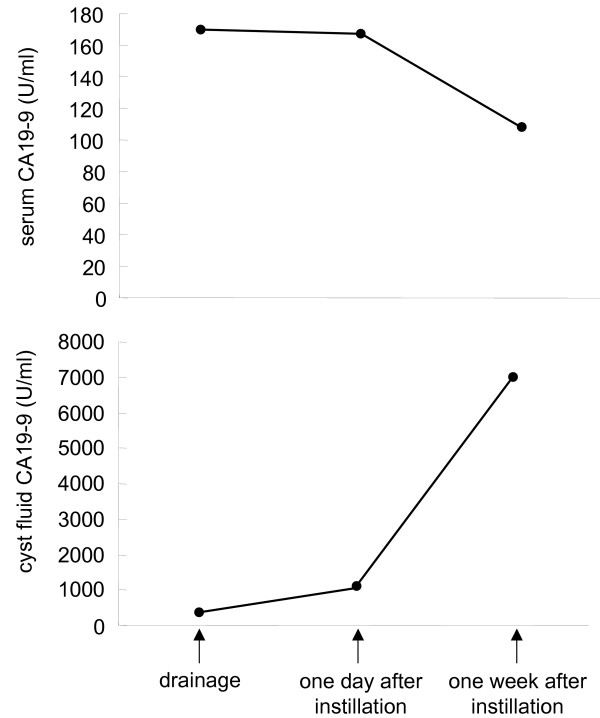
Changes in serum and cyst fluid CA19-9 levels before and after the intracystic instillation of minocycline hydrochloride.

In addition, we performed percutaneous treatment of the hepatic cyst by drainage and sclerotherapy using minocycline hydrochloride. Briefly, after local anesthesia of the puncture site with 1% mepivacaine hydrochloride, a 20-cm long, 22-gauge needle was passed into the cyst under real-time ultrasonic guidance with a 3.5 MHz convex transducer. After a pig tail catheter had been inserted into the cyst, the cystic fluid was aspirated. Bacteria, neoplastic cells, and parasites were not detected in the cystic fluid, however, CA19-9 levels were elevated in hepatic cyst fluid (371 U/ml). At 3 days after drainage, her fever and serum CRP level (4.1 mg/dl) were remarkably decreased; this also supported the diagnosis of an infected hepatic cyst. 400 mg of minocycline hydrochloride was dissolved in 50 ml of saline, and this minocycline solution was injected into the cyst using the drainage catheter. The drain was closed, and was opened 24 hours after intracystic instillation of minocycline hydrochloride. To determine the contribution of the hepatic cyst to the CA19-9 levels, we measured serum and cyst fluid CA19-9 levels before, 1 day after and 1 week after intracystic instillation of minocycline hydrochloride. After intracystic instillation, necrotic cells in the cyst were drained, and the cyst totally collapsed after 1 week (Figure 1B). Cyst fluid CA19-9 levels increased remarkably after the minocycline instillation, while serum CA19-9 levels decreased (Figure 2).

## Discussion

Imaging modalities such as CT and ultrasound are highly accurate for diagnosing simple hepatic cysts, however, the distinction between cystadenoma and a simple hepatic cyst complicated by intracystic hemorrhage has been reported to be difficult [3]. The measurement of serum and cyst fluid CA19-9 levels has been reported to be helpful in distinguishing between a hemorrhagic simple cyst and cystadenoma or cystadenocarcinoma [4,5]. However, serum and cyst fluid CA19-9 levels were increased in our patient with a simple hepatic cyst before treatment, challenging this suggestion. Elevated serum and cyst fluid CA19-9 levels in our patient before treatment may be due to an infected hepatic cyst. Yoshida *et al. *also observed elevated serum and cyst fluid CA19-9 levels in a patient with an infected hepatic cyst [2], and in a patient with a simple hepatic cyst complicated with intracystic hemorrhage [6]. Further, elevated serum CA19-9 levels were found in a patient with an inflammatory pseudotumor of the liver [7]. Sawabu *et al. *reported that serum CA19-9 levels in patients with cholelithiasis complicated by cholangitis frequently showed markedly high values, and serum CA19-9 levels were rapidly decreased and normalized by amelioration of inflammation [8], indicating a significant association between inflammation of the hepatobiliary system and CA19-9 levels. In our patient, cyst fluid CA19-9 levels were elevated concomitant with increased flow of necrotic cyst wall cells after the minocycline instillation, suggesting that CA19-9 may originate from the hepatic cyst wall cells. Inflammation, including infection and the minocycline instillation-induced tissue injury may induce necrosis of the hepatic cyst wall cells, and may consequently increase serum and cyst fluid CA19-9 levels. This study revealed that the treatment of a simple hepatic cyst by instillation of minocycline hydrochloride significantly decreased the serum CA19-9 levels, suggesting that serum CA19-9 may also originate from the hepatic cyst.

## Conclusion

To our knowledge, our study is the first report to reveal a significant influence of intracystic instillation of minocycline hydrochloride on serum and cyst fluid CA19-9 levels in a patient with a simple hepatic cyst. Further clinical studies are needed in a large number of patients.

## Competing interests

The authors declare that they have no competing interests.

## Authors' contributions

HY treated the patient, analyzed and interpreted the patient data, and was a contributor in writing the manuscript. NT advised on the format and design and assisted in providing a critical appraisal of the manuscript. Both authors have reviewed and approved the final manuscript.

## Consent

Written informed consent was obtained from the patient for the publication of this case report and any accompanying images. A copy of the written consent is available for review by the Editor-in-Chief of this journal.

## References

[B1] Blonski WC Campbell MS Faust T Metz DC Successful aspiration and ethanol sclerosis of a large, symptomatic, simple liver cyst: case presentation and review of the literature World J Gastroenterol 2006 12 2949 2954 16718826 10.3748/wjg.v12.i18.2949 PMC4087818

[B2] Yoshida H Onda M Tajiri T Mamada Y Taniai N Mineta S Hirakata A Futami R Arima Y Inoue M Hatta S Kishimoto A Infected hepatic cyst Hepatogastroenterology 2003 50 507 509 12749258

[B3] Kitajima Y Okayama Y Hirai M Hayashi K Imai H Okamoto T Aoki S Akita S Gotoh K Ohara H Nomura T Joh T Yokoyama Y Itoh M Intracystic hemorrhage of a simple liver cyst mimicking a biliary cystadenocarcinoma J Gastroenterol 2003 38 190 193 10.1007/s005350300032 12640536

[B4] Lee JH Chen DR Pang SC Lai YS Mucinous biliary cystadenoma with mesenchymal stroma: expressions of CA 19-9 and carcinoembryonic antigen in serum and cystic fluid J Gastroenterol 1996 31 732 736 10.1007/BF02347626 8887044

[B5] Horsmans Y Laka A Gigot JF Geubel AP Serum and cystic fluid CA 19-9 determinations as a diagnostic help in liver cysts of uncertain nature Liver 1996 16 255 257 8877996 10.1111/j.1600-0676.1996.tb00738.x

[B6] Yoshida H Onda M Tajiri T Mamada Y Taniai N Uchida E Arima Y Akimaru K Yamashita K Intracystic hemorrhage of a simple hepatic cyst Hepatogastroenterology 2002 49 1095 1097 12143211

[B7] Ogawa T Yokoi H Kawarada Y A case of inflammatory pseudotumor of the liver causing elevated serum CA19-9 levels Am J Gastroenterol 1998 93 2551 2555 10.1111/j.1572-0241.1998.00717.x 9860425

[B8] Sawabu N Takemori Y Toya D Yoneshima M Kidani H Satomura Y Ohta H Hattori N Factors affecting serum levels of CA 19-9 with special reference to benign hepatobiliary and pancreatic diseases Gastroenterol Jpn 1986 21 491 498 3465664 10.1007/BF02774633

